# A Survey on Attitude, Awareness, and Knowledge of Patients Regarding the Use of Dental Implants at a Swiss University Clinic

**DOI:** 10.3390/dj11070165

**Published:** 2023-07-05

**Authors:** Adib Al-Haj Husain, Olivia De Cicco, Bernd Stadlinger, Fabienne Andrina Bosshard, Valérie Schmidt, Mutlu Özcan, Silvio Valdec

**Affiliations:** 1 Clinic of Cranio-Maxillofacial and Oral Surgery, Center of Dental Medicine, University of Zurich, 8032 Zurich, Switzerland; adib.al-hajhusain@zzm.uzh.ch (A.A.-H.H.); olivia.decicco@uzh.ch (O.D.C.); bernd.stadlinger@zzm.uzh.ch (B.S.); fabienne.bosshard@zzm.uzh.ch (F.A.B.); valerie.schmidt@zzm.uzh.ch (V.S.); 2 Division of Dental Biomaterials, Clinic for Reconstructive Dentistry, Center of Dental Medicine, University of Zurich, 8032 Zurich, Switzerland; mutluozcan@hotmail.com; 3 Clinic of Masticatory Disorders and Dental Biomaterials, Center of Dental Medicine, University of Zurich, 8032 Zurich, Switzerland

**Keywords:** dental implant, awareness, attitude, knowledge, questionnaire, tooth replacement, oral surgery

## Abstract

Even though restoring missing teeth and oral tissue with dental implants is perceived by most patients as a positive experience, patients lack access to evidence-based information about different treatment options. In order to provide more accurate information for public dental education in Switzerland and to compare it worldwide, this descriptive cross-sectional survey-based study assessed pre-operative attitudes, awareness, and knowledge of patients. A total of 160 patients with indication for tooth extraction were selected randomly from clinical routine between August 2022 and February 2023. Statistical analysis was performed including the chi-square test based on a significance level of 0.05. The results confirm that most patients (78%) were aware of implants as a treatment option for replacing missing teeth and consider them a prioritized solution (79%). Their primary sources of information are dentists (59%), the Internet (50%), relatives and friends (40%). The majority of patients (84%) wanted the surgical procedure to be performed by a board-certified clinical specialist expecting high functional and aesthetic outcomes. Low levels of knowledge could be observed regarding postoperative care, functionality, and clinical performance of implants. This survey-based study revealed a positive attitude and an acceptable level of awareness and knowledge regarding the use of dental implants.

## 1. Introduction

As in most high-income countries, there is a demographic shift toward an increasingly aging population due to increased life expectancy; medical healthcare is facing medical, economic, and social challenges. The aging population and increasing demand for medical services also pose daily challenges for dentistry in prevention, rehabilitation, and patient-specific care management [1].

Tooth loss is a severe life event associated with the impairment of several essential oral functions, especially eating and speaking. It is also often associated with various psychological and physical side effects on different aspects of oral health-related quality of life (OHRQoL), which, in addition to an unsatisfactory appearance, can significantly limit affected patients in their daily activities [2,3]. Modern oral implantology aims to meet the increasing expectations of aesthetically aware patients. It is therefore accompanied by a growing demand for implant-supported fixed prostheses that improve subjective oral health and patient satisfaction [4,5]. Various reports show that dental implants, developed initially to treat edentulous patients, can positively affect individuals with missing teeth by improving denture retention, stability, chewing efficiency, and patient self-confidence and satisfaction [6,7,8]. Even though restoring missing teeth and oral tissue with dental implants is perceived by most patients as a positive experience, patients lack access to evidence-based information about different treatment options, as data published on the Internet and especially on social media do not accurately reflect scientific evidence [9]. However, the widespread information from various sources such as the Internet, television, books, relatives, or medical professionals is being reflected in the increasing popularity and use of dental implants as a prosthetic treatment option within the dental field. 

From a medical, economic, and psycho-social perspective, dental implants are considered the best long-term treatment solution for replacing single or multiple missing teeth [10,11]. However, the high cost of implants can be considered a significant barrier for patients to choose this treatment option. Moreover, the “novelty” of this treatment approach and the associated high costs may lead to unrealistic expectations, which can negatively affect patient satisfaction with the treatment outcome [8]. Hence, several surveys have been conducted to determine patient awareness regarding the use of dental implants, identifying varying levels of knowledge, sources of information, and requirement for information in different study populations worldwide [12,13,14,15,16].

In general, awareness and interest (79–95%) in oral implant therapy have increased worldwide over the past two decades with study populations in high-income industrialized countries such as the United States [12] or Austria [13], as well as in developing countries such as Saudi Arabia [14,16] and India [15], generally showing favorable interest in this treatment option. However, there are regional differences in patient awareness regarding expectations for perioperative dental implant treatment and follow-up care, required oral hygiene, and financial costs based on the widely disseminated information sources in the media.

In order to provide more general and accurate evidence-based information for public dental education in Switzerland about dental implant therapy and to compare it worldwide, this descriptive cross-sectional survey-based study aimed to determine the attitudes, awareness, sources of information, and level of knowledge of patients at a Swiss university clinic regarding the use of dental implants to replace missing teeth.

## 2. Materials and Methods

### 2.1. Study Design and Sample Selection

This descriptive cross-sectional survey-based study was conducted at the Clinic of Cranio-Maxillofacial and Oral Surgery, Center of Dental Medicine, University of Zurich, Zurich, Switzerland. It included adult patients referred for surgical tooth extraction either by a general practitioner or on their own initiative. A self-explanatory online questionnaire was designed to assess the attitudes, awareness, sources of information, and level of knowledge of patients in Switzerland regarding dental implant therapy for missing teeth. Data was collected between August 2022 and February 2023 via an online platform survey.

The following criteria were required to be included: (1) indication for tooth extraction; (2) male and female patients older than 16 years; (3) no previous dental implant surgery in the last 5 years. Exclusion criteria were: (1) very old (>85 years) or uncooperative patients; (2) mentally or physically disabled patients.

To minimize selection bias in the participant recruitment process, several measures were implemented. First, every second patient with an indication for extraction was invited to participate in the study. Second, the study included a diverse population of patients, irrespective of their familiarity with prosthetic replacement procedures, including those who had undergone dental implant surgery and those who had not. Third, the study was conducted at multiple sites (the emergency station of the department and the regular clinic routine) to ensure a broad and representative sample. Furthermore, the study procedure was designed in a manner that neither patients nor physicians needed to know if the questionnaire had been completed or was yet to be completed.

### 2.2. Questionnaire

The questionnaire included 30 questions divided into two sections that captured the following: demographic data, socioeconomic status, level of education, oral hygiene, as well as knowledge, attitude, awareness, and source of information on dental implants. The development process of the questionnaire followed a systematic process with an analysis of the existing literature. Thus, this questionnaire was slightly modified but mainly in line with the questionnaires already used in the literature [12,13,14,15,16]. This initial step was followed by thorough review by the clinic’s experts in the field of investigation to identify any potential issues such as duplicate, confusing, or double-leading questions. The questionnaire was prepared in German and English to ensure the best possible understanding of the questions. To assess its validity and reliability, the questionnaire underwent specific pre-tests involving ten carefully selected subjects who represented the target population under investigation. Furthermore, responding to every question was not mandatory since patients were given the choice to skip certain questions if they preferred not to answer them. The power analysis was estimated using existing literature [8]. The questionnaire is included as Supplementary Material.

### 2.3. Statistical Analysis

The data processing and analysis involved utilizing statistical methods to examine the frequency distribution of the data. Certain answers were encoded as dichotomous variables, representing yes/no responses, while others were encoded as categorical variables when multiple choices were involved. Given the nature of this survey-based study, descriptive statistics was calculated for the majority of the questions. In addition to descriptive statistics, *t*-test and chi-square test were used to compare categorical data in a contingency table, with the level of significance level set at *p* = 0.05. All data obtained during the data collection phase were transferred to an Excel spreadsheet (Microsoft Excel 2020, Microsoft Corporation, Redmond, WA, USA). Then, the responses were carefully double-checked and cleaned by two researchers (A.A.H. and S.V.). The data were afterwards analysed using IBM SPSS Statistics software (version 27.0, IBM Corp. Armonk, NY, USA).

### 2.4. Ethical Consideration

This study was conducted according to the guidelines of the Declaration of Helsinki and its later revised ethical standards. Each study participant who took part in this study provided written informed consent. No specific ethical approval was required for this survey-based study with respect to local ethics committee regulations.

## 3. Results

One hundred and sixty patients participated in the study. Among the 160 subjects, 53% (N = 84) were male and 47% (N = 74) were female, with a mean age of 38.39 ± 16.65 (median: 32; age range: 16–77). The participation rate (people who accessed and participated in the survey) was 76.9%, and the completion rate (people who participated and completed the survey) was 80.6%. Most participants have a university degree or high school diploma. The average participation time was 4:56 min (truncated mean). More detailed demographic information on the included patients can be found in Table 1. Please consider, that patients were given the choice to skip certain questions if they preferred not to answer them.

**Figure 1:**
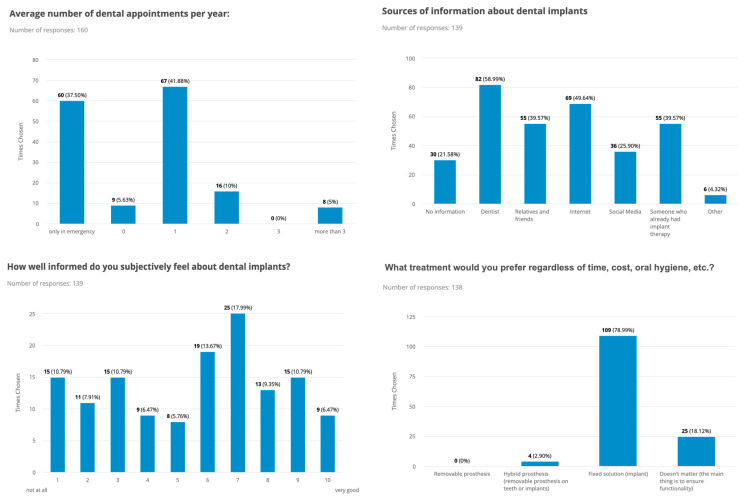
Key data on the number of dental visits, sources of information and knowledge about dental implants, and the preferred treatment option of the included patients are presented in bar charts.

Most study participants had one dental appointment per year (42%) and one dental hygiene appointment per year (52%), while 38% visited their dentist only in emergencies. They rated their oral hygiene as good (78% with a score ≥ 7 out of 10) and mainly used the following oral hygiene products: manual (65%) and electric (61%) toothbrushes, dental floss (52%) and rinsing solution (47%). Of participants 31% had gum problems that required treatment. There was a significant difference between men and women (*p* ≤ 0.001). The average annual budget for all dental costs was up to CHF 1000 for 67% of the participants. Of the study participants 74% had at least a tooth extraction and 60% had multiple tooth extractions. In terms of knowledge about the use of dental implants to restore a missing tooth, 78% of the study participants were aware of dental implants as a treatment option, of which 64% felt subjectively well to very well informed about dental implants. Thereby no significant difference could be observed between males and females (*p* = 0.68). The main sources of information about dental implants were the dentist (59%), followed by the Internet (50%), people who previously had implant treatment or friends and relatives (both 40%), and social media (26%). Of those questioned 63% showed high interest (score ≥ 8 out of 10) in implant restoration for a tooth gap or in case of tooth loss, while 14% already had had dental implant surgery in the past (>5 years ago) (Figure 1).

**Figure 2:**
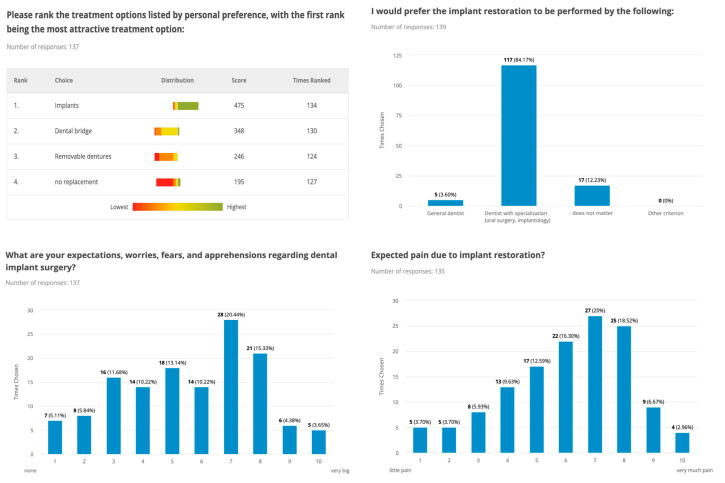
Key data on attitudes and awareness about dental implants among included patients are presented in bar graphs. X-Axis: numeric rating scale ranging from 1 to 10.

**Figure 3:**
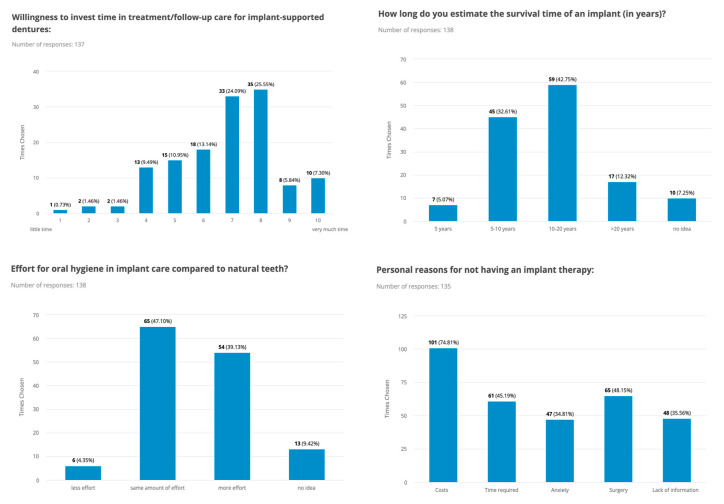
Key data on knowledge of dental implants and their postoperative care, as well as survival and personal reasons for not undergoing implant therapy in the included patients, are presented in bar charts.

Regardless of factors that might influence treatment planning, such as time, cost, or oral hygiene, patients comparably preferred dental implant therapy in 79% of cases, followed by dental bridges, removable dentures, and no tooth replacement. Reasons against implant surgery were cost (75%), surgical procedure (48%), time (45%), lack of information (36%), and fear of the procedure (35%). The majority of the patients (84%) desired the dental implant procedure to be performed by a board-certified clinical specialist (oral surgery, implantology), and they indicated that they would be willing to pay a higher treatment charge for the same procedure in Switzerland (45%—up to CHF 3000) than abroad (62%—up to CHF 2000). Regarding these aspects no significant difference could be observed between males and females (*p* > 0.05) (Figure 2 and Figure 3).

The majority of the study participants had high expectations of surgical therapy, with both functional (57%) and aesthetic (46%) outcomes being very important to them, with female gender and young age being factors that that correlated with a higher value on aesthetic outcomes. Hence, there was a significant difference between men and women (*p* ≤ 0.001). Concerning oral hygiene and the care of implants, most study participants were willing to invest time in the follow-up of implant-supported prostheses and expected the same (47%) or more (39%) effort, aware that perioperative pain may occur, and 43% expected implants to last 10–20 years. More detailed information on attitude, awareness, and knowledge about dental implants among the included patients is shown in Figure 1, Figure 2 and Figure 3.

## 4. Discussion

The present survey-based study investigated patient attitudes, awareness, and knowledge at a Swiss university clinic regarding the potential use of dental implants as a therapeutic option for replacing missing teeth. The majority of this Swiss cohort (78%) was aware of dental implants as a prosthetic treatment option, with many reporting that they felt subjectively well-informed about this procedure. Awareness of oral health varied between sociodemographic cohorts, with higher awareness among female compared to male study participants and individuals with higher than lower education or income levels. Nearly 42% consulted their dentist at least once a year, and around half consulted a dental hygienist, confirming trends from previous studies in developed and high-income European countries such as Switzerland [17]. 

Regarding the knowledge of the use of dental implants to restore masticatory function and aesthetics, the results of this study reflect and confirm the increasing popularity and knowledge of this treatment approach in most developed countries and also in developing countries [14,16,18]. However, there is still a significant difference compared to middle- and low-income countries, as the lower level of awareness in low-income countries may be strongly influenced by socioeconomic status and education levels [15]. 

The results presented in this study show that almost all study participants had multiple sources of information, with dentists being the main source of information (59%), closely followed by the Internet (50%), friends and relatives in general and especially those who had already had dental implant treatment (both 40%) and social media (26%). Other studies showed different and heterogeneous results compared to the data published in the literature. In the Netherlands, 52% of respondents received their information first from friends, while 36% received it from their general dentist [19]. In Japan, only 20% sought information from their general dentist [20], while in a survey in the U.S., only 17% first sought information about dental implants from their dentist, with the media and friends playing a more important role [12]. However, other studies confirm the results of our study that the most common source of information was the dentist or relatives and friends [14,21,22]. Thus, despite the heterogeneity regarding this aspect in the literature, it can be concluded that there is a lack of evidence-based information, confusion, and misunderstanding, especially about biological, technical, and aesthetic considerations, as many patients obtain information from relatives and friends, the Internet, and social media, giving the impression that implant therapy is always possible and the best treatment option. In addition, Tepper et al. showed that public media, such as magazines, television, and social media, play an essential role in spreading the idea of everlasting implants that do not require special care and hygiene [13]. This ideal conception is also reflected in the results of the present study: 43% assume that implants will last at least 10 to 20 years, while 61% estimated the postoperative oral hygiene effort to be unchanged, less, or had no idea at all, reflecting the low level of knowledge about the functionality and clinical performance of dental implants. 

As previously mentioned, dental implants are increasingly becoming the focus of interest for patients and dentists as it is a growing treatment modality associated with a high success rate [23]. However, interdisciplinary evidence-based decision-making must always consider various procedure- and patient-specific factors. The results of this study confirm that the financial aspect contributes significantly to the choice of therapeutic strategy. This observation aligns with the findings of Zimmer et al. and Tepper et al., where up to 76% of interviewees reported the high cost of implants as the most compelling argument against their use [12,13]. In addition, the relationship between treatment costs and the patient’s income was highlighted in our study, revealing that patients with higher incomes were more inclined to choose dental implants as their initial and preferred treatment option, regardless of the associated effective costs. However, if study participants had the opportunity to choose the therapeutic approach independently of medical or economic factors, most patients would prefer implant-supported fixed dentures. The average annual budget for all dental costs was up to CHF 1000 for 67% of the participants, which does not cover the price of dental implant treatment, even if the participants are willing to pay more if the procedure is performed in Switzerland. Nevertheless, despite the high costs, implant therapy is positively related to improving dental health, comfort, speech, mastication, and thus, the quality of life in general [23]. These results are difficult to compare with other data in the literature as they are highly dependent on the health care system, the health insurance system, and the real gross domestic product. Two other survey studies performed reported that most of patients had acceptable to good knowledge and awareness as well as a positive attitude towards using dental as a treatment option for replacing missing teeth [16,24], which agrees with the results of this current survey.

In all industrialized countries, however, the high price of treatment is always a factor in the treatment decision. At the same time, it is more affordable for people compared to low-income countries where this treatment option is usually only available to wealthier sections of the population. However, in the choice of practitioners, the wish for experienced and specialized professionals as well as high aesthetics and functionality is expressed by most patients, which confirms the results published earlier, both in low-income and high-income countries [13,23]. However, the trend towards increased use of implants by less experienced and non-specialized dentists in implant therapy could be a risk factor for the success rate of the surgical procedure and the acceptance of implant treatment [23]. While the increasing number of dentists performing this surgical procedure is of interest to the industry, the long-term survival of implants in high-risk cases should be monitored, which also points to the need for dental implant education [23].

Several limitations should be considered when interpreting the results of this cross-sectional survey-based study. First, the participants included in this study were all recruited from the surgical department of a university clinic. Consequently, most had planned an extraction or even a more complex surgical procedure. While this is precisely our target population, they obviously do not reflect the opinion of the general public, thus limiting the external validity of our study. For more general conclusions, larger studies with a more heterogeneous population are needed to confirm the identified trends with higher reliability and validity. Second, this study used self-reported information that may be influenced by various evaluator or systematic biases. Hence, replacing or supplementing the data assessment with a more objective evaluation method could also provide more reliable data. The participants in this study were comparatively younger, which may affect various aspects of the assessment such as attitude, access to the Internet and social media, or financial capabilities. The survey did not include any questions regarding post operational procedures and possible complications, which could be considered in future surveys.

## 5. Conclusions

This survey-based study showed that almost all patients were aware of dental implants, reflecting the increasing popularity of this treatment approach. However, there is a lack of evidence-based information on multi-criteria decision-making, especially on biological, technical, and aesthetic considerations, as many patients obtain information from relatives and friends, the Internet, and social media, which gives the perception that implant therapy is always possible and the most accurate treatment option. In addition, the financial aspect plays a major role in the selection of a therapeutic approach, while in the choice of practitioner, the desire for experienced and specialized professionals is expressed by most patients. Given the innovations in imaging technologies, implant manufacturing, and the implementation of state-of-the-art in digital workflows, dental implant surgery might play an increasingly important role in dental rehabilitation. Therefore, patients should be provided with more evidence-based data, indication possibilities along with possible complications.

## Figures and Tables

**Table 1:** Demographic characteristics of the study participants.

**Age**	**Year**	**N**	**%**
	16–27	53	34%
	28–38	40	25%
	39–49	21	13%
	≥50	43	27%
**Gender**	Male	74	47%
	Female	84	53%
**Educational Level**	Compulsory education	11	7%
	Apprenticeship diploma	47	31%
	High School	34	22%
	University degree	61	40%
**Occupation**	self-employed/freelancer/ executive	18	11%
	white-collar worker/civil servants	63	39%
	blue-collar workers	17	11%
	persons working in agriculture	3	2%
	pupils/students	29	18%
	housewives	4	3%
	retirees	14	9%
	others/no response	12	8%
**Estimated Total Annual Income**	<30,000.-	49	32%
	>37,000–79,000.-	45	30%
	>80,000.-	49	32%

## Data Availability

The data presented in this study are available on request from the corresponding author. The data are not publicly available.

## Supplementary Materials

The following supporting information can be downloaded at: https://www.mdpi.com/article/10.3390/dj11070165/s1.
