# Supplementary material for: A Survey on Attitude, Awareness, and Knowledge of Patients Regarding the Use of Dental Implants at a Swiss University Clinic
Source: Dent J (Basel). 2023 Jul 5;11(7):165. doi: 10.3390/dj11070165 (PMC10378414; doi:10.3390/dj11070165)
Supplement: Supplementary file 1 [file dentistry-11-00165-s001.zip › dentistry-2435596-supplementary.pdf]

Universität Zürich[UZH]

Zentrum für Zahnmedizin
Klinik für Mund-, Kiefer- und Gesichtschirurgie
Poliklinik für Oralchirurgie

Direktor a.i. Oralchirurgie: Prof. Dr. Thomas Attin
Plattenstrasse 11
8032 Zürich
Telefon 044 634 32 90
Telefax 044 634 43 28
zzm.oc@zzm.uzh.ch
www.zzm.uzh.ch

Prof. Dr. Dr. Bernd Stadlinger
Stv.Klinikdirektor
Telefon +41 44 634 32 90
Telefax +41 44 634 43 28

Dr. Silvio Valdec
Leiter Klinikbetrieb
Telefon +41 44 634 32 90
Telefax +41 44 634 43 28

"A Survey-Based Analysis of Attitude, Awareness, and Knowledge of Patients regarding the Use of Dental Implants at a Swiss University Clinic"

General information:

Gender:______________________ Age: ______________________

Occupation: ______________________

Educational Background: ▢ Compulsory education ▢ Apprenticeship diploma ▢ High School ▢ University degree

Estimated annual income:
▢ <30'000.- ▢ >37'000-79'000.- ▢ >80'000.-

Do you have any dental insurance?
▢ yes ▢ no If yes, which franchise: ___________________________________

Average number of dental appointments per year: ▢ Only in emergency ▢ 0 ▢ 1 ▢ 2 ▢ 3 ▢ 4 ▢ 5 ▢ >5

Average number of dental hygiene appointments per year: ▢ 0 ▢ 1 ▢ 2 ▢ 3 ▢ >3

Annual budget for all dental costs: ▢ till 1000.- ▢ 1000-2000.- ▢ 2000-3000.- ▢ over 3000.-

How would you rate your own oral hygiene?
▢ 1 ▢ 2 ▢ 3 ▢ 4 ▢ 5 ▢ 6 ▢ 7 ▢ 8 ▢ 9 ▢ 10
1 bad 10 very good

What oral hygiene products do you use? (multiple selection possible)
▢ Electric toothbrush ▢ Manual toothbrush ▢ Rinsing solutions ▢ Dental floss ▢ Interdental brushes

Have you ever had gum problem that required treatment? ▢ yes (current) ▢ yes (treated) ▢ no

How many tooth extractions have you had?

▢ 1 ▢ 2 ▢ 3 ▢ 4 ▢ 5 ▢ 6 ▢ 7 ▢ 8 ▢ 9 ▢ >10

Attitude, awareness, and knowledge about implants:

Have you ever had a dental implant treatment?
▢ yes ▢ no If, yes how many: ___________________________

Interested in an implant restoration for a tooth gap or in case of toothloss:
▢ 1 ▢ 2 ▢ 3 ▢ 4 ▢ 5 ▢ 6 ▢ 7 ▢ 8 ▢ 9 ▢ 10
1 no interest *10 great interest*

Sources of information about dental implants (multiple selection possible):
▢ No information ▢ Dentist ▢ Friends ▢ Internet ▢ Social Media
▢ Someone who already had implant therapy ▢ Other:
____

How well informed do you subjectively feel about dental implants?
▢ 1 ▢ 2 ▢ 3 ▢ 4 ▢ 5 ▢ 6 ▢ 7 ▢ 8 ▢ 9 ▢ 10
1 not at all *10 very good*
What do you prefer for treatment regardless of time, cost, oral hygiene, etc.?
▢ Removable prosthesis ▢ Hybrid prosthesis (removable prosthesis on teeth or implants) ▢ Fixed solution (implant) ▢ Doesn't matter (the main thing is to ensure functionality)

Sort the mentioned treatment options with 1 (best) – 4 (worst) according to personal priority:
▢ Dental bridge _______________________ ▢ Removable dentures _________________________
▢ Implants _______________________ ▢ no replacement _________________________

I would prefer the implant restoration to be performed by the following:
▢ General dentist
▢ Dentist with specialization (oral surgery, implantology).
▢ does not play a role
▢ Other criterion: ______________________________

Payment willingness for an implant-supported dental prosthesis in Switzerland:
▢ 2000.- ▢ 3000.- ▢ 4000.- ▢ 5000.- ▢ egal

Payment willingness for an implant-supported dental prosthesis in other countries:
▢ 2000.- ▢ 3000.- ▢ 4000.- ▢ 5000.- ▢ egal

What are your expectations, worries, fears, and apprehensions regarding dental implant surgery?
▢ 1 ▢ 2 ▢ 3 ▢ 4 ▢ 5 ▢ 6 ▢ 7 ▢ 8 ▢ 9 ▢ 10
1 none *10 very big*

Expected pain due to implant restoration?
▢ 1 ▢ 2 ▢ 3 ▢ 4 ▢ 5 ▢ 6 ▢ 7 ▢ 8 ▢ 9 ▢ 10
1 little pain Schmerzen *10 very much pain*

Willingness to invest time in treatment/follow-up care for implant-supported dentures:
▢ 1 ▢ 2 ▢ 3 ▢ 4 ▢ 5 ▢ 6 ▢ 7 ▢ 8 ▢ 9 ▢ 10
1 little time *10 very much time*

How long do you estimate the survival time of an implant (in years)?
▢ 5 years ▢ 5-10 years ▢ 10-20 years ▢ >20 years ▢ no idea

Effort for oral hygiene in implant care compared to natural teeth?
▢ less effort ▢ same amount of effort ▢ more effort ▢ no idea

Personal reasons for not having an implant therapy (multiple selection possible):

▢ Costs ▢ Time required ▢ Anxiety ▢ Surgery ▢ Lack of information ▢ other

How important is the functional result of an implant-supported denture for you?
▢ 1 ▢ 2 ▢ 3 ▢ 4 ▢ 5 ▢ 6 ▢ 7 ▢ 8 ▢ 9 ▢ 10
1 not important *10 very important*

How important is the esthetic result of an implant-supported denture for you?
▢ 1 ▢ 2 ▢ 3 ▢ 4 ▢ 5 ▢ 6 ▢ 7 ▢ 8 ▢ 9 ▢ 10
1 not important *10 very important*

Thank you for participating in the survey! All data collected in this survey will be treated confidentially and evaluated anonymously.
